# Impact of chronic unpredicted mild stress-induced depression on repaglinide fate via glucocorticoid signaling pathway

**DOI:** 10.18632/oncotarget.17874

**Published:** 2017-05-15

**Authors:** Hongyan Wei, Ting Zhou, Boyu Tan, Lei Zhang, Mingming Li, Zhijun Xiao, Feng Xu

**Affiliations:** ^1^ Fengxian Hospital, Southern Medical University, Shanghai, China; ^2^ Hunan Provincial People's Hospital, Hunan Normal University, Changsha, China; ^3^ Joint Research Center for Translation Medicine, East China Normal University, Shanghai, China

**Keywords:** chronic unpredicted mild stress(CUMS), depression, repaglinide, drug-metabolizing enzymes (DMEs), glucocorticoid and adrenergic signaling pathway

## Abstract

Chronic unpredicted mild stress (CUMS)-induced depression could alter the pharmacokinetics of many drugs in rats, however, the underlying mechanism is not clear. In this work we studied the pharmacokinetics of repaglinide, and explored the role of glucocorticoid and adrenergic signaling pathway in regulating drug metabolizing enzymes (DMEs) in GK rats and BRL 3A cells. The plasma cortisol and epinephrine levels were increased, meanwhile the pharmacokinetics of repaglinide were altered significantly in depression model rats. Forty-nine genes in liver of model rats displayed significant difference comparing to control rats. The differentially expressed genes enriched in the drug metabolism and steroid hormone biosynthesis pathway significantly, and *Nr1i3* matched 335 connectivity genes. CAR and Ugt1a1 protein expression were enhanced significantly in liver of model rats. The mRNA expression of *Ugt1a1* and *Nr1i2* were increased 2 and 4 times respectively with dexamethasone (DEX) and 8-Br-cAMP co-treatment in BRL 3A cells. The protein expression of PXR was up-regulated, too. However, RU486 reversed the up-regulated effect. The adrenergic receptor agonists had little impact on the DMEs in BRL 3A. Our data suggested that CUMS-induced depression might up-regulate DMEs expression via glucocorticoid signaling pathway, and accelerate the fate of the repaglinide in spontaneous diabetes rats.

## INTRODUCTION

Stress is a constant factor in modern life and has become one of the most important problems affecting human health in our society [1–4]. Long-term stress may disturb the homeostasis, and lead to psychological diseases such as major depressive disorder [5–7]. The hypothalamic-pituitary-adrenal (HPA) axis with the end-point release of corticosterone into the circulation plays a role in mediating the neuroendocrine response to stress [8]. Once given with depressive stress, concentrations of corticotropin-releasing hormone (CRH) are usually elevated, and then declined with the administration of antidepressants [9]. Evidence showed that maternal deprivation stress may modify the expression of many cytochrome P-450 (CYP450) genes, thus affecting the efficacy and toxicity of relevant drugs [10]. The impact of stress on drug metabolism is stress-specific, isozyme-specific, gender-specific, and species-specific [11].

Currently more documents demonstrated that comorbidity with depression in diabetes might influence hypoglycemic agent efficacy in diabetic patients [12]. Repaglinide, a new prandial glucose regulator, is metabolized dominantly by CYP2C8 and partially by CYP3A4 in human beings [13]. If CYP2C8 is inhibited due to various reasons, it will be replaced by CYP3A4 [14]. CYP3A4 is regulated by many transcription factors, among which the pregnane X receptor /steroid and xenobiotic receptor (PXR/SXR, *Nr1i2*) have been identified as the most critical. The genetic polymorphism of PXR had impact on the pharmacokinetics and pharmacodynamics of repaglinide in healthy Chinese volunteers, and showed that subjects with genotype of −298G/G and 11193C/C in PXR have a decreased elimination rate of CYP3A4/2C8 [15]. Our previous work found that chronic unpredicted mild stress (CUMS)-induced depression could alter the pharmacokinetics of many drugs in rats [16–19]. However, the molecular mechanisms underlying the stress and drug metabolism change are not fully understood. We herein explored the pharmacokinetics of repaglinide in depression model rats first, and then focused on the role of glucocorticoid and adrenergic signaling pathway in regulating drug-metabolizing enzymes (DMEs) expression in model rats.

## RESULTS

### Establishment of CUMS-induced depression in GK rats

**Figure 1:**
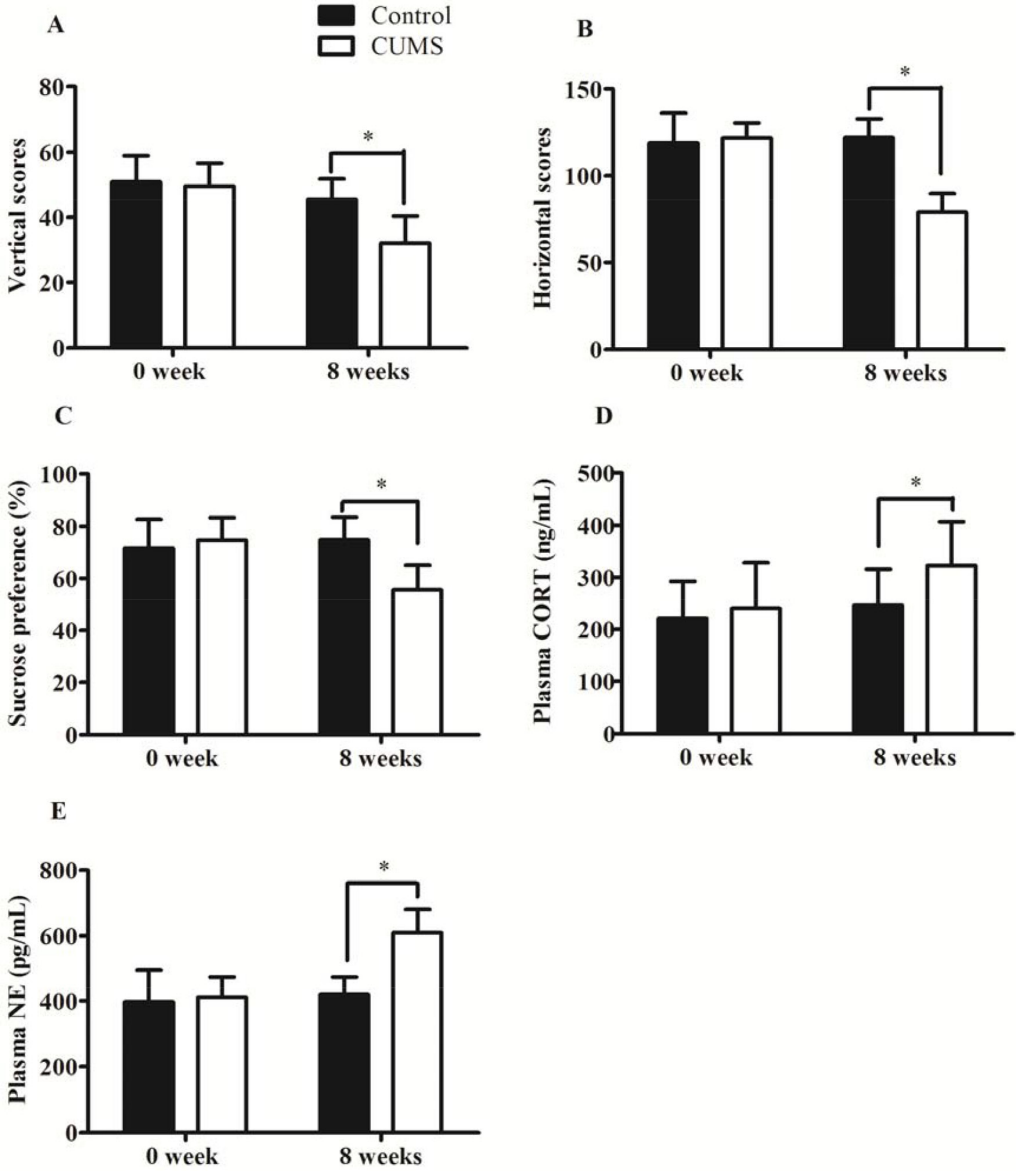
Validation of CUMS-induced depression model in GK rats (**A**) Vertical scores; (**B**) Horizontal scores; (**C**) Sucrose Preference Tests; (**D**) Plasma CORT concentration; (**E**) Plasma NE concentration. The data are presented as mean ± SD (*n* = 10). *p < 0.05, compared with control group.

As shown in Figure 1, no significant differences were found between the two groups prior to model establishment. However, after 8 weeks’ stress, the rats in the CUMS depression group displayed depressive-like behaviors. Open-field test showed that the vertical and horizontal scores in the CUMS depression group significantly decreased from 50.83 ± 8.04 to 32.08 ± 8.36 (*p* < 0.01) and 121.75 ± 8.82 to 79.08 ± 10.55 (*p* < 0.01), respectively. The sucrose preference values in the CUMS depression group significantly decreased from 71.63 ± 11.08 to 55.53 ± 9.55 (*p* < 0.01). At the same time the plasma cortisol (CORT) and epinephrine (NE) concentration in CUMS depression group were significantly increased about 30% (240.83 ± 87.49 to 322.67 ± 84.11, *p* < 0.05) and 45% (411.14 ± 61.31 to 609.75 ± 71.21, *p* < 0.05), respectively. No change was existed within control group. These results confirmed CUMS-induced depression rat model was established successfully.

### Pharmacokinetics disturbance of repaglinide in GK depression model rats

**Figure 2:**
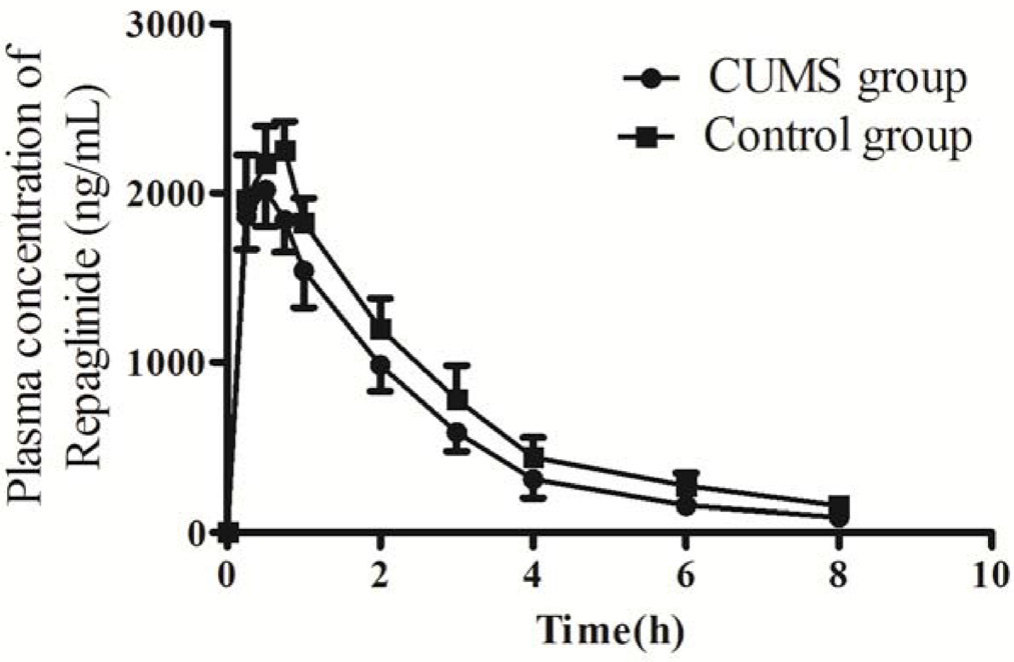
The plasma concentration-time curves of repaglinide in GK rats The data were presented as mean ± SD (*n* = 10).

CUMS-induced depression on repaglinide pharmacokinetics in rats were presents in Figure 2 and Table 1. CUMS-induced depression accelerated the fate of repaglinde: speeding T_1/2_ by 17.4 % (from 2.53 ± 0.33 h to 2.09 ± 0.46, *p* < 0.05), T_max_ by 26.8% ( from 0.71 ± 0.10 h to 0.52 ± 0.07 h, *p* < 0.05), and decreasing C_max_ by 10.7% (from 2263.46 ± 187.18 ng/ml to 2020.56 ± 208.31 ng/ml, *p* < 0.05), AUC_0-∞_ by 22.3% (from 6685.46 ± 983.24 ng/ml·h to 5194.02 ± 801.19 ng/ml·h, *p* < 0.05) and MRT_0-∞_ by 18% (from 3.00 ± 0.17 h to 2.46 ± 0.12 h, *p* < 0.05).

**Table 1:** The main pharmacokinetic parameters of repaglinide in plasma of GK rats

Parameters	CUMS group	Control group
T_1/2_ (h)	2.09 ± 0.46*	2.53 ± 0.33
T_max_ (h)	0.52 ± 0.07*	0.71 ± 0.10
C_max_ (ng/mL)	2020.56 ± 208.31*	2263.46 ± 187.18
AUC_0-∞_ (ng/mL·h)	5194.02 ± 801.19*	6685.46 ± 983.24
MRT_0-∞_ (h)	2.46 ± 0.12*	3.00 ± 0.17

*p < 0.05, compared with control group (*n* = 10, mean ± SD).

### Analysis and confirmation of differentially expressed genes in liver tissue of GK rats

**Figure 3:**
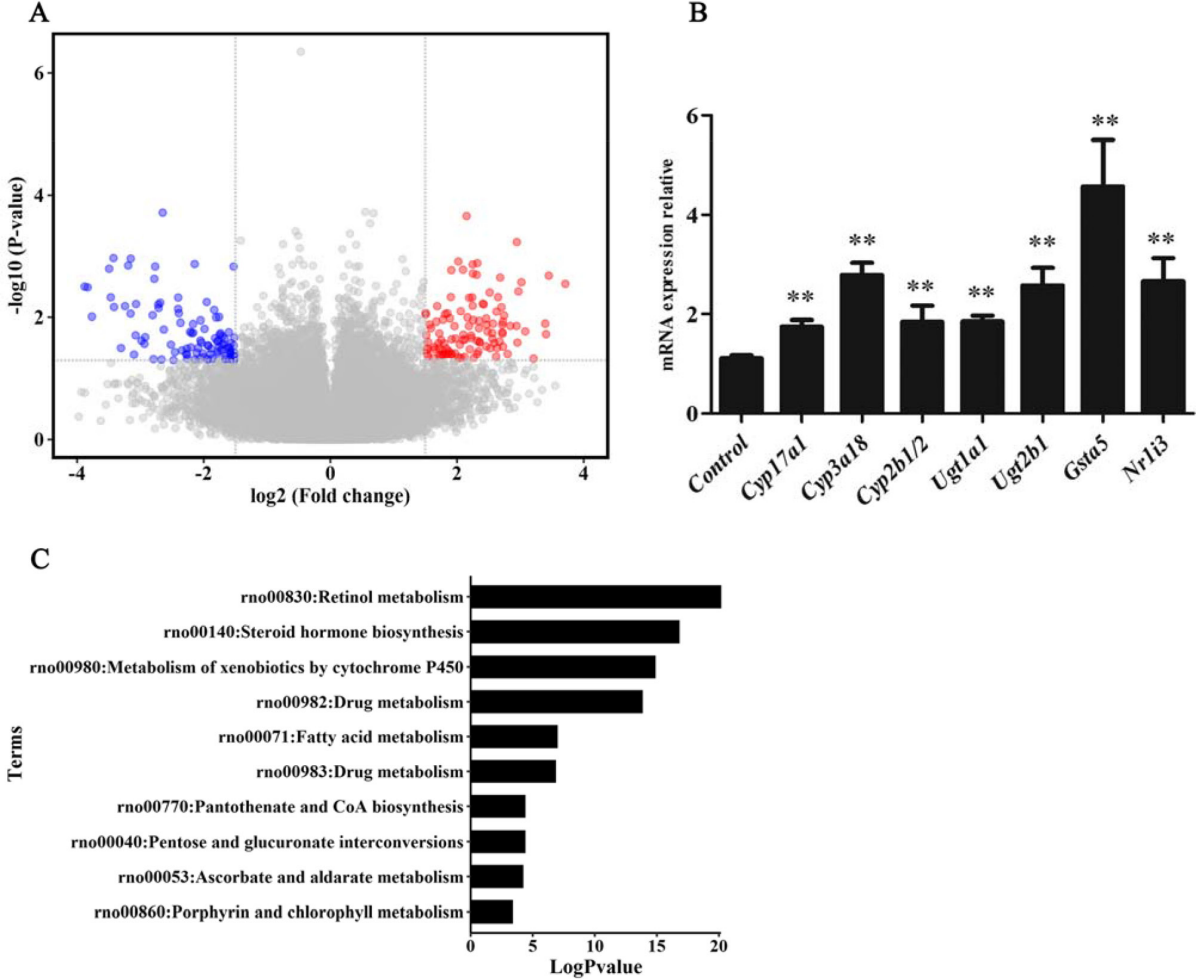
mRNA expression levels and KEGG pathway analyses (**A**) The volcano plot image showed the mRNA expression levels of microarray in CUMS group compared with control. Black dots: equally expressed mRNAs between CUMS group compared and control (0.5 ≤ Fold Change ≤ 1.5); red dots: mRNAs were overexpressed in CUMS group compared with control (*P*-values < 0.05, Fold Change ≥ 1.5); blue dots: mRNAs in CUMS group were down-expressed compared to control (*P*-values < 0.05, Fold Change ≤ 0.5). Fold changes of these mRNAs in CUMS group compared with control are shown as mean ± SD. (**B**) Relative expression of differentially expressed genes(DEGs) using qRT-PCR confirmation(normalized to controls). Data are presented as the mean ± SD, *n* = 6, **p < 0.01, compared with control. (**C**) KEGG pathway enrichment analysis of the up-regulated DEGs.

**Figure 4:**
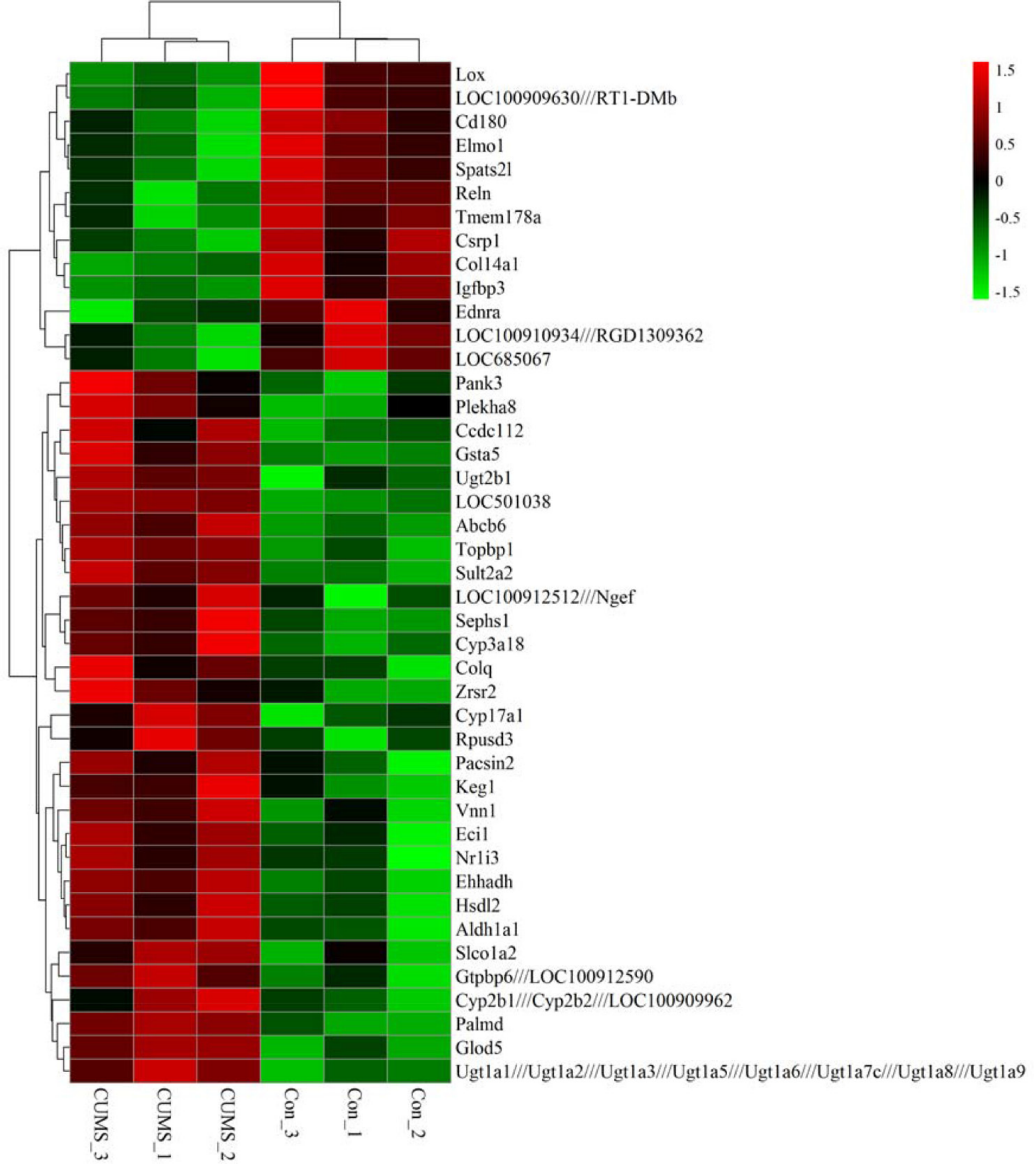
Hierarchical clustering heat map of differentially expressed genes in liver tissue of GK rats between CUMS and control group Up-regulation was indicated by red, down-regulation by green. Clusters of genes showing similarity of the expression patterns were identified based on Euclidian distances.

**Figure 5:**
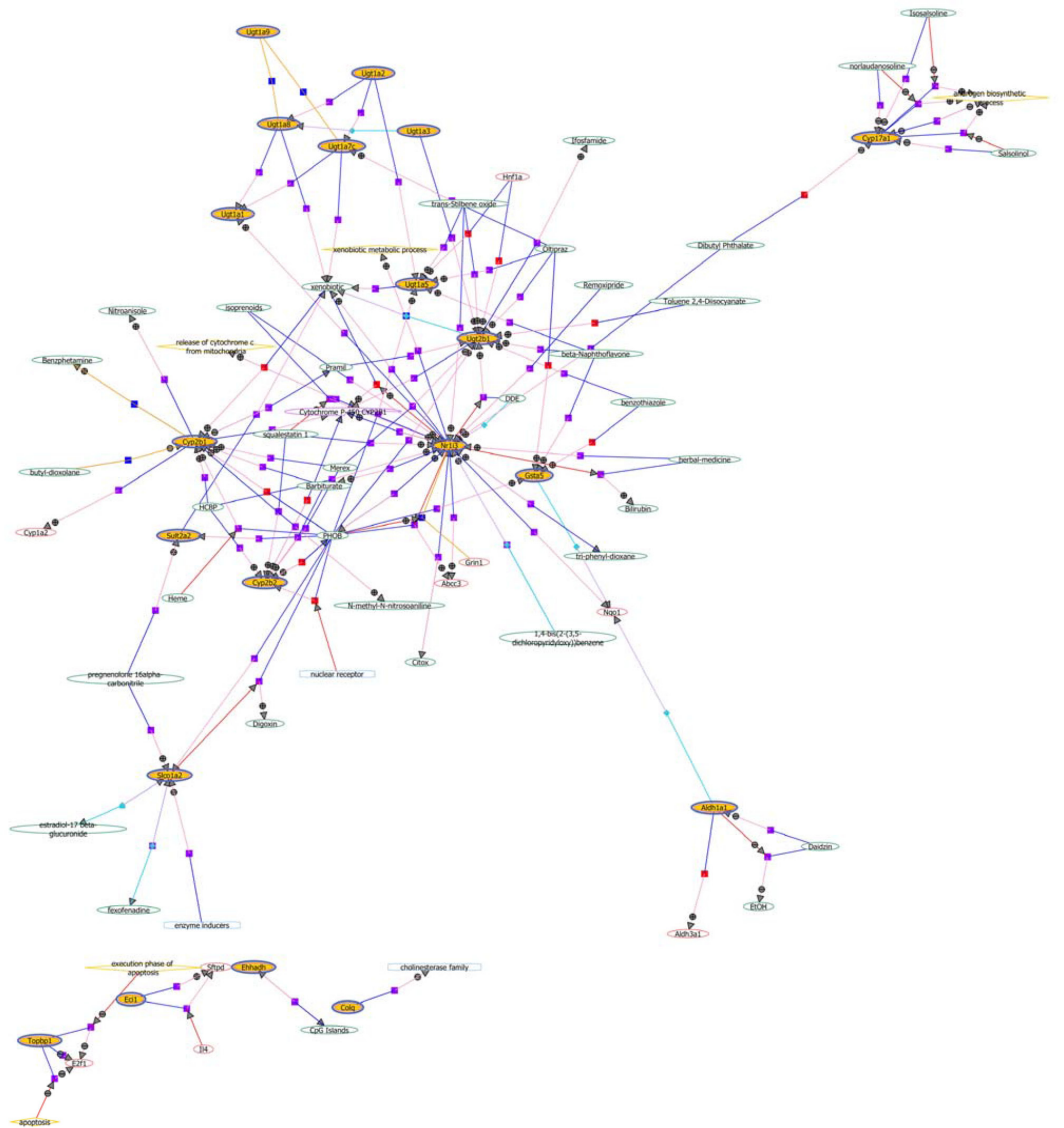
The network of up-regulated differentially expressed genes in liver tissue of GK rats The yellow circle indicated the differentially expressed genes, and the arrows indicated the regulation directions. The plus sign denoted the positive regulation, while the minus sign denoted the negative regulation.

To show mRNA expression profile in CUMS-induced depression GK rats, we used a stringency cutoff to identify significantly differently mRNAs (*P* < 0.05, Fold Change ≥ 1.5 or ≤ 0.5) and two-dimensional hierarchical clustering 3.0 to represent expression profiles between samples. The GeneChip results illustrated that a greater number of differentially expressed probes were observed between the two groups, which matched 49 differentially expressed genes (DEGs). Among them, 13 DEGs were down-regulated, and 36 were up-regulated in depression rats (Figure 3A). The hierarchical clustering of DEGs was visualized as a heatmap (Figure 4). Based on the GeneSpring software analysis the up-regulated DEGs were interconnected and formed gene expression networks. *Nr1i3* matched 335 connectivity, which regulated the *Ugt1a1, Ugt2b1, Cyp3a18* and others genes expression and function (Figure 5 and Table 2).

**Table 2:** The network of the up-regulated differentially expressed genes

Probe Set ID	Gene Symbol	Gene Title	*P* Value	Fold Change	Global Connectivity
1395976_at	*Plekha8*	pleckstrin homology domain containing, family A (phosphoinositide binding specific) member 8	0.043	1.524	1
1387924_at	*Ngef*	ephexin-1-like///neuronal guanine nucleotide exchange factor	0.047	1.76	4
1370592_at	*Keg1*	kidney expressed gene 1	0.036	1.77	4
1389253_at	*Vnn1*	vanin 1	0.030	2.00	6
1368068_a_at	*Pacsin2*	protein kinase C and casein kinase substrate in neurons 2	0.046	1.56	6
1368475_at	*Colq*	collagen-like tail subunit (single strand of homotrimer) of asymmetric acetylcholinesterase	0.047	1.85	12
1381958_at	*Topbp1*	topoisomerase (DNA) II binding protein 1	0.005	1.62	15
1398307_at	*Cyp3a18*	cytochrome P450, family 3, subfamily a, polypeptide 18	0.028	1.50	16
1367659_s_at	*Eci1*	enoyl-CoA delta isomerase 1	0.033	1.64	19
1370613_s_at	*Ugt1a8*	UDP glucuronosyltransferase 1 family, polypeptide A 8	0.004	1.51	19
1387936_at	*Sult2a2*	sulfotransferase family 2A, dehydroepiandrosterone (DHEA)-preferring, member 2	0.003	2.74	20
1370613_s_at	*Ugt1a7c*	UDP glucuronosyltransferase 1 family, polypeptide A7C	0.004	1.51	20
1371089_at	*Gsta5*	glutathione S-transferase Yc2 subunit	0.028	2.77	43
1387123_at	*Cyp17a1*	cytochrome P450, family 17, subfamily a, polypeptide 1	0.032	2.90	49
1368283_at	*Ehhadh*	enoyl-CoA, hydratase/3-hydroxyacyl CoA dehydrogenase	0.008	1.58	69
1370613_s_at	*Ugt1a5*	UDP glucuronosyltransferase 1 family, polypeptide A5	0.004	1.51	71
1371076_at	*Cyp2b2*	cytochrome P450, family 2, subfamily b, polypeptide 2	0.049	1.90	100
1387093_at	*Slco1a2*	solute carrier organic anion transporter family, member 1A2	0.044	1.53	102
1371076_at	*Cyp2b1*	cytochrome P450, family 2, subfamily b, polypeptide 1	0.049	1.90	141
1370698_at	*Ugt2b1*	UDP glucuronosyltransferase 2 family, polypeptide B1	0.035	1.60	150
1387022_at	*Aldh1a1*	aldehyde dehydrogenase 1 family, member A1	0.016	2.08	152
1368797_at	*Nr1i3*	nuclear receptor subfamily 1, group I, member 3	0.043	2.06	335

To determine which pathway might be involved in CUMS-induced depression, KEGG pathway analysis was used to authenticate pathways and understand biological functions of significantly differentially expressed genes. The result indicated that the up-regulated DEGs were enriched in 10 pathways, including the drug metabolism, steroid hormone biosynthesis, and so on (Figure 3C and Table 3). Steroid hormone biosynthesis (rno00140, FDR = 0.007) and Drug metabolism (rno00982, FDR = 0.058) were significantly enriched pathways. Five genes, namely *Cyp3a18, Ugt1a1, Ugt2b1,Cyp2b1/2* and *Gsta5*, were included in drug-metabolism pathway. Additionally, *Cyp17a1* instead of *Gsta5*, and other four genes, were also enriched in steroid hormone biosynthesis pathway.

**Table 3:** KEGG pathway enrichment analysis of the up-regulated DEGs

KEGG ID	KEGG term	Count	Log *P* value	FDR	Genes
rno00830	Retinol metabolism	5	2.02E + 01	7.31E-04	*Ugt1a1, Aldh1a1, Cyp2b1/2, Cyp3a18, Ugt2b1*
rno00140	Steroid hormone biosynthesis	5	1.68E + 01	0.007	*Ugt1a1, Cyp3a18, Cyp17a1, Ugt2b1, Sult2a2*
rno00980	Metabolism of xenobiotics by cytochrome P450	5	1.49E + 01	0.028	*Gsta5, Ugt1a1, Cyp2b1/2, Cyp3a18, Ugt2b1*
rno00982	Drug metabolism	5	1.39E + 01	0.058	*Gsta5, Ugt1a1, Cyp2b1, Cyp3a18, Ugt2b1*
rno00071	Fatty acid metabolism	2	7.00E + 00	6.517	*Ehhadh, Eci1*
rno00983	Drug metabolism	3	6.87E + 00	7.113	*Ugt1a1, Cyp3a18, Ugt2b1*
rno00770	Pantothenate and CoA biosynthesis	2	4.40E + 00	34.041	*Vnn1, Pank3*
rno00040	Pentose and glucuronate interconversions	2	4.40E + 00	34.041	*Ugt1a1, Ugt2b1*
rno00053	Ascorbate and aldarate metabolism	2	4.23E + 00	37.606	*Ugt1a1, Ugt2b1*
rno00860	Porphyrin and chlorophyll metabolism	2	3.39E + 00	57.737	*Ugt1a1, Ugt2b1*

KEGG, Kyoto Encyclopedia of Genes and Genomes; FDR, false discovery rate; DEGs, differentially expressed genes.

These DEGs relevant to drug-metabolism and steroid hormone biosynthesis in the liver tissue of GK rats were validated by quantitative real-time polymerase chain reaction (qRT-PCR). The result showed that *Cyp17a1, Cyp3a18, Cyp2b1/2, Ugt1a1, Ugt*2b1, *Gsta5*, and *Nr1i3* were increased approximately 2–4 folds in liver tissue of model rats compared with control rats (Figure 3B). The relative foldchanges detected by qRT-PCR were consistent with the microarray results, indicating the dependability of our microarray platform.

### Effect of glucocorticoids and adrenergic pathway on drug metabolizing-related genes expression in BRL 3A cells

**Figure 6:**
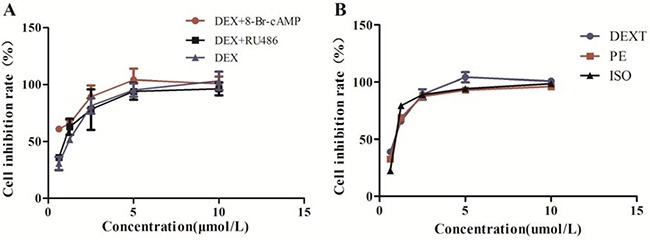
Cell viability (**A**) DEX alone, DEX combination with 8-Br-cAMP or RU486 treated BRL 3A for 48 hr. (**B**) DEXT, PE and ISO treated BRL 3A for 48 hr. Data were presented as the mean ± SD (normalized to controls), *n* = 6, *p < 0.05 vs control.

CCK assay showed that DEX, DEXT, PE, ISO of 1 μmol/L does not affect BRL 3A cell viability. DEX of 1 μmol/L was set as combination concentration as co-treated with 8-Br-cAMP or RU486, respectively in the following study (Figure 6).

**Figure 7:**
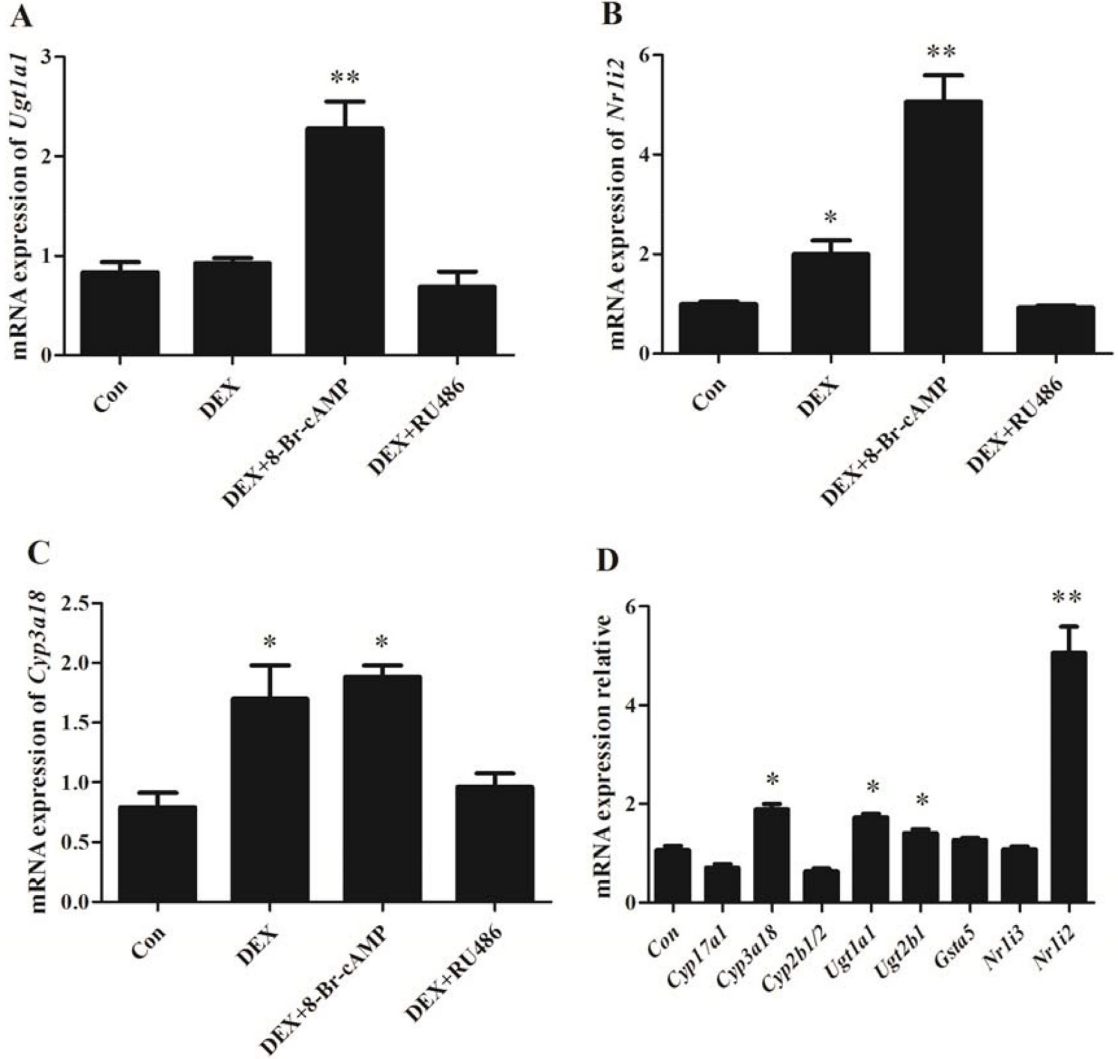
The mRNA expression in BRL 3A cells with DEX, RU486, 8-Br-cAMP treatment for 48 hr (**A**) *Ugt1a1*; (**B**) *Nr1i2*; (**C**) *Cyp3a18*; (**D**) Differentially expressed genes relevant to drug-metabolism and steroid hormone biosynthesis. Data are presented as the mean ± SD of the relative mRNA level (normalized to controls), *n* = 3, *p < 0.05, **p < 0.01 vs control.

**Figure 8:**
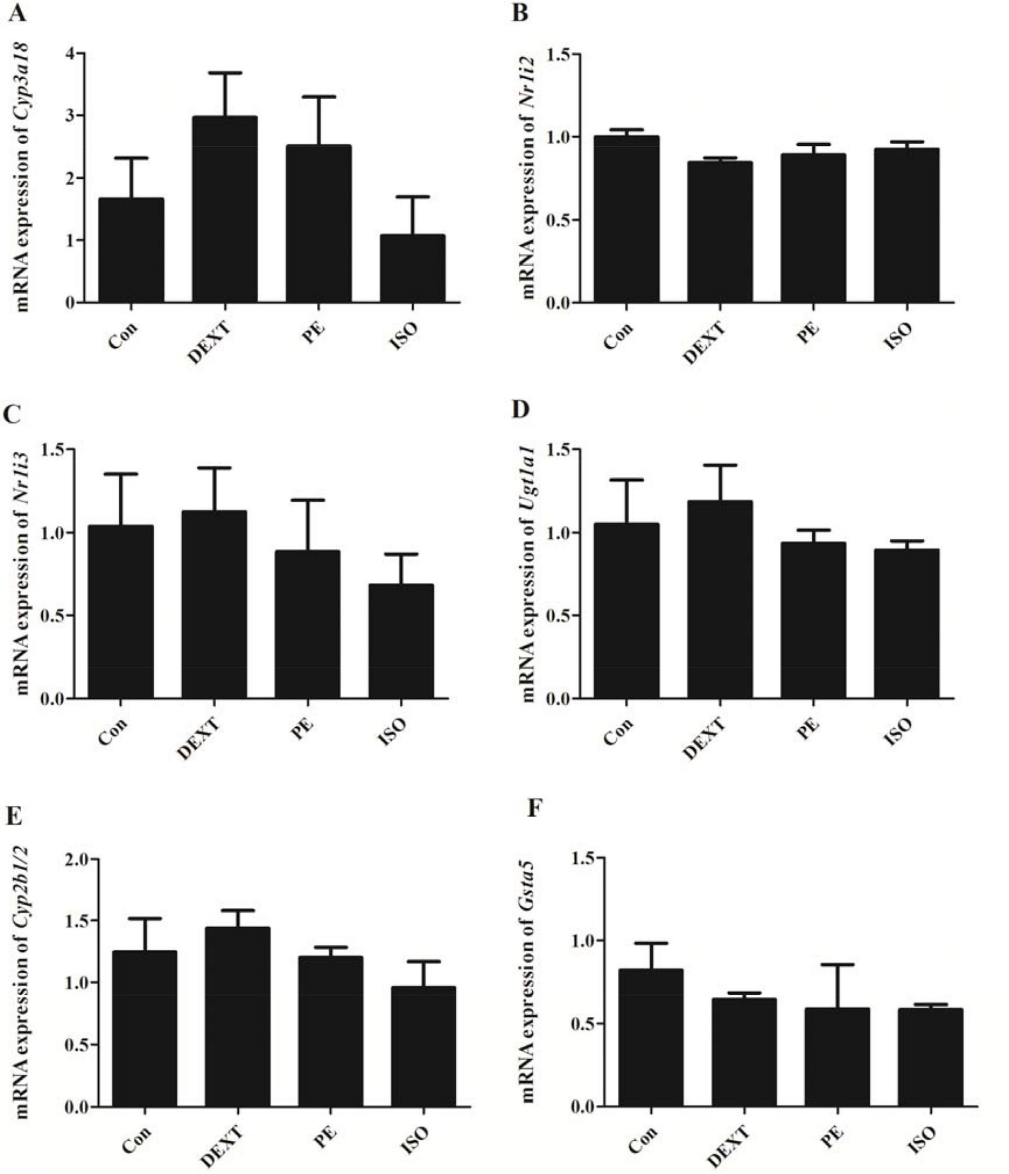
The mRNA expression in BRL 3A cells with DEXT, PE and ISO treatment for 48 hr (**A**) *Cyp3a18*; (**B**) *Nr1i2*; (**C**)*Nr1i3*; (**D**) *Ugt1a1;* (**E**) *Cyp2b1/2*; (**F**) *Gsta5*. Data are presented as the mean ± SD of the relative mRNA level (normalized to controls), *n* = 3.

*In vitro* cell test displayed that the mRNA expression of *Ugt1a1* and *Cyp3a18* were enhanced significantly with DEX alone and DEX plus 8-Br-cAMP co-treatment for 48hr in BRL 3A cells (Figure 7). Specifically, *Nr1i2* was up-regulated 2 times with DEX alone treatment and 4 times with DEX plus 8-Br-cAMP co-treatment. RU486 reversed the effect of DEX. However *Cyp17a1* and *Cyp2b1/2* appeared down-regulated. The α-adrenergic receptor agonists DEXT and PE enhanced Cyp3a18 expression moderately, but the difference was not significant. In addition, β-receptor agonists ISO had little effect on *Cyp3a18, Ugt1a1, Ugt2b1, Gsta5, Nr1i2* and *Nr1i3* expression (Figure 8).

### Alteration of drug metabolizing enzymes both in liver tissue of GK rats and in BRL 3A cells

**Figure 9:**
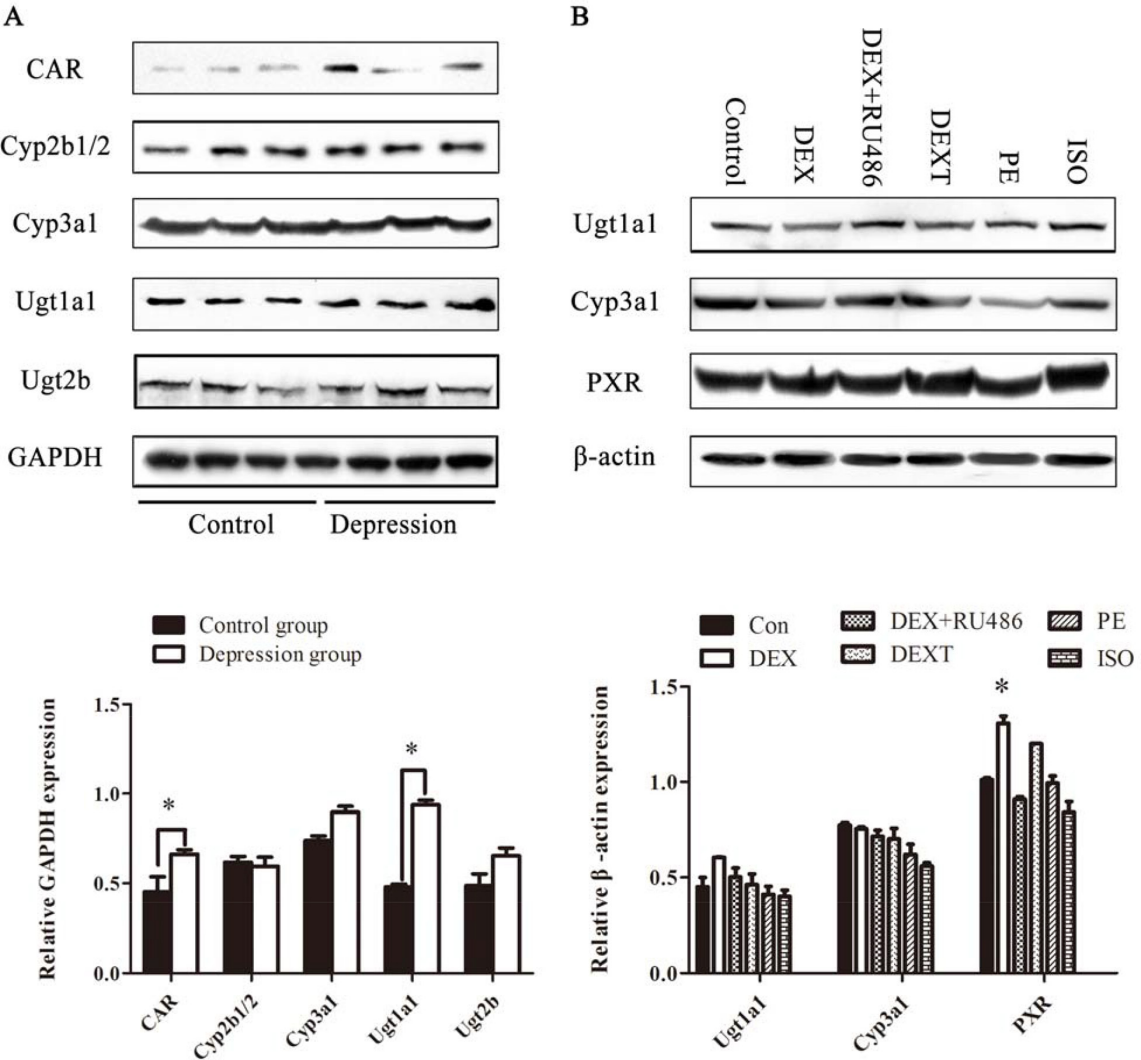
The protein expression of drug metabolizing enzymes (**A**) The protein expression of CAR, Cyp2b1/2, Cyp3a1, Ugt1a1, Ugt2b in GK rats’ liver. (**B**) The protein expression of Ugt1a1, Cyp3a1, PXR in BRL 3A. Data are presented as the mean ± SD of the relative protein (normalized to controls), *n* = 3, *p < 0.05 vs control.

To further investigate protein expression of the interested genes, Western blot was applied both in liver tissue of GK rats and in BRL 3A cells. As shown in Figure 9 the protein expression levels of Cyp3a1 was enhanced moderately, but Ugt1a1 was enhanced significantly in liver tissue of GK rats (*p* < 0.05). The CAR was increased significantly in liver tissue of GK model rats, meanwhile PXR was increased significantly with DEX plus 8-Br-cAMP treatment for 48hr in BRL 3A cells (*p* < 0.05). Adrenergic receptor antagonists including DEXT, PE and ISO didn't alter the drug metabolizing enzymes expression.

## DISCUSSION

Various physiological and pathological factors such as age, sex, and individual genetic background can affect drug metabolism. Recently evidences showed that psychological factors also influence the drug metabolism process [16–19]. We herein established the CUMS induced-depression model with GK rats. We noted that stress hormone cortisol level increased significantly and repaglinide pharmacokinetics changed in model rats. Leblond et al. found that stress hormone might regulate the expression of CYP2C11, CYP 3A1/2 in rats with chronic renal failure [20]. Therefore we surmised that pharmacokinetic change in depression model rats might be consequence of DMEs expression alters associated with hormone stress.

In order to explore the underlying mechanism, we compared the gene expression profiles of liver tissue of depression model rats to that of control rats. The results indicated that mRNA expression of *Cyp17a1, Cyp3a18, Cyp2b1/2, Gsta5, Ugt1a1* and *Ugt2b1* were up-regulated significantly in model group rats. These differentially expressed genes (DEGs) that met the thresholds yielded and enriched in KEGG pathways of drug metabolism-enzymes and steroid hormone biosynthesis. *Nr1i3* was the central node of up-regulated DEGs Using GeneSpring software analysis, which influenced the most of other genes expression. The mRNA expression of *Cyp17a1, Cyp3a18, Cyp2b1/2, Gsta5, Ugt1a1, Ugt2b1* and *Nr1i3* in gene chip was confirmed by qRT-PCR. At same time the protein expression of Cyp3a1, Cyp2b1/2, and Ugt2b were increased slightly in model rats compared with control rats, but Ugt1a1 and CAR were up-regulated significantly.

The biosynthesis of cortisol is dependent on the activity of microsomal CYP17α-hydroxylase/17,20-lyase [21] meanwhile steroid hormone enhances the CYP450 expression under stress [22]. In this study *Cyp17a1* mRNA expression was up-regulated significantly in liver tissue of model rats, and cortisol was in increased in model rats, demonstrated the delicate interaction among drug-metabolism enzymes expression, stress hormone and stress in model rats. UDP-glucuronosyl transferases (UGTs) are major phase II enzyme family in the liver micro some [23]. Among them Ugt1a1 detoxifies bilirubin neurotoxicity by conjugating with glucuronic acid, therefore plays a critical role in the detoxification and excretion of endogenous and exogenous lipophilic compounds [24]. Species and tissue/cell-dependent regulation of UGT expression by ligand-activated transcription factors is often involved in the regulation of homeostasis [25]. Ugt1a1 expression was up-regulated significantly in this study, suggested that Ugt1a1 participate in substrates metabolism to cope with stress. The transcription factor *Nr1i3*, encoding orphan nuclear receptor CAR, regulates gene encoding DMEs and transporters in livers of rats [26]. Both mRNA expression of *Nr1i3* and protein expression of CAR were consistently up-regulated in depression model rats in this study. Besides GeneSpring analysis results indicated *Nr1i3* regulates Ugt1a1 and other DMEs expression.

In liver tissue of model rats KEGG pathway analysis highlighted significant enrichment in drug metabolism-enzymes and steroid hormone biosynthesis pathway. To further study the role of steroid hormone in regulating the differential expression of DMEs induced by stress, rat liver cell line of BRL 3A was treated with DEX. We noted that it was *Nr1i2* (rather than *Nr1i3*) that increased twice as treated with DEX for 48 hr, while glucocorticoid receptor antagonist RU486 reversed this stimulatory effect. When BRL 3A cells were co-treated with DEX and 8-Br-cAMP(analog of cAMP) for 48 hr, both *Ugt1a1* and *Nr1i2* were up-regulated significantly (*Nr1i2* was up-regulated almost 4 folds), along with *Nrli2* encoding PXR was up-regulated. Since both glucocorticoids and cAMP stimulates corticotropin releasing hormone (CRH) promoter through the cAMP response element (CRE) [21], the synergistic stimulatory effect of DEX and 8-Br-cAMP on *Ugt1a1* and *Nr1i2* might be mediated through the CRE. Over the past two decades, differential expression of DMEs induced by stress rose as an important pharmacology hot issue. Coordinated regulation of DMEs and transporters are mediated by a number of transcription factors [27] including CAR and PXR [28]. The transcriptional pathways that are activated in response to stress via CAR and PXR may be better explained the *Ugt1a1* expression in this study.

Although epinephrine plasma level increased significantly in depression model rats, it seems that epinephrine has little-to-no effect on DMEs expression *in vitro* experiment. As BRL3A cells were treated with the selective adrenergic agonists DEXT and PE for 48 hr, *Cyp3a18* expression was only moderately up-regulated without any significant difference. Similarly *Cyp2b1/2, Ugt1a1*, *Gsta5, Nr1i2* and *Nr1i3* expression also changed a little. The protein expression of Ugt1a1, Cyp3a1 and PXR were broadly unchanged, too. Besides, β-adrenergic agonist ISO had no effect on the drug metabolism-related gene/protein expression. The mechanism of the little-to-no effect of adrenergic receptor agonist *in vitro* assay is not clear. Adrenergic receptors are expressed in the central nervous system and peripheral nervous system involving in the regulation of various physiological functions [29, 30]. Glucocorticoids are the major regulators of CYP450, and their release are affected by the adrenergic signaling pathway [31]. Stress might directly promote the expression of CYP3A, CYP2C and CYP2D via the adrenergic pathway, or indirectly regulate the expression of CYP450 through insulin pathway [32]. The *in vitro* little-to-no effect of adrenergic receptor agonists in BRL 3A cells suggested that adrenergic pathway might be dominantly related with the release of glucocorticoids *in vivo*.

Our data suggested that CUMS-induced depression might up-regulate DMEs expression via glucocorticoid signaling pathway, and accelerate the fate of the repaglinide in spontaneous diabetes rats. Nuclear transcription factor *Nr1i3* and *Nr1i2* were specific in regulating DMEs genes under stress via glucocorticoid hormone.

## MATERIALS AND METHODS

### Materials

TRIzol Reagent was from Life Technologies (Carlsbad, CA, USA). RNeasy micro kit and RNase-Free DNase Set were bought from QIAGEN (GmBH, Germany). Gene Chip 3’IVT Express Kit, GeneChip^®^ Hybridization, Wash and Stain Kit were from Affymetrix (Santa Clara, CA, USA). Dulbecco minimum essential medium (DMEM), phosphate buffer saline (PBS), fetal bovine serum (FBS), penicillin-streptomycin solution, and sodium dodecyl sulfate (SDS) were obtained from Gibco (Life Technologies, USA). Rat cortisol (CORT) and epinephrine (NE) ELISA Kit were purchased from CUSABIO (CUSABIO, USA). The RNA Extraction Kit (Code No.RR036A), PrimeScript TM RT Master Mix (Code No.RR047A), SYBR Premix Ex Taq TM (Code No.RR420A) were purchased from TaKaRa Bio Inc (Dalian, China). The repaglinide, nateglinide, dexamethasone(DEX), 8-Br-cAMP, RU486, dexmedetomidine (DEXT), phenylephrine (PE), isoprenaline (ISO), dimethyl sulphoxide (DMSO) were purchased from Sigma (St. Louis, MO, USA), The Cyp3a1, Cyp2b1/2, Ugt1a1, Ugt2b, CAR, PXR, GAPDH, β-actin primary antibody and the horseradish peroxidase conjugated goat anti-rabbit antibody were bought from Abcam (CA, USA), Pierce ECL Western Blotting Substrate from Thermo Scientific (CA, USA). Nuclear extracts (RIPA), BCA Protein Assay Kit, 5×loading buffer, Prestained Protein Molecular Weight Marker were bought from Beyotime Biotech Reagents (Shanghai, China ). The Pure Nitrocellulose (NC) Blotting Membrane was bought from Bio-Rad Laboratories (Hercules, CA, USA). All other chemicals were of analytical grade and purchased from Shanghai Biotech (Shanghai, China).

### Establishment of CUMS-induced depression model

Five weeks old male GK rats were purchased from Shanghai SLAC Laboratory Animal Co., Ltd. (Animal Quality Certificate: 2007000562918), were kept in the Laboratory of Animal Center, East China Normal University, Shanghai (Animal Experiment License: SYXK 2010–0094). Rats were kept in the cage for 7 weeks in an SPF-grade lab until the emergence of diabetes (with blood glucose level ≥ 11 mmol/L). Then the diabetic rats were randomly divided into two groups: control group and depression model group (*n* = 10 per group). The depression model group rat was fed alone in a cage for 8 weeks with food and water ad libitum. Each rat was given one kind of stresses daily. The stresses included activity restriction (in bottle, 1 hr), hot water swimming (45°C, 5 min), cold water swimming (4°C, 5 min), clip tail (1 min), cages tilting (45°C, 24 hr), cage horizontal shaking (10 min), damp padding (24 hr), noise interference (10 min), and day/night inversion (24 hr). Each stress was used 5–6 times randomly but not consecutively to avoid rat's prediction. The control group rats were normally fed for 8 weeks with food and water ad libitum without any stress.

The open-field test was performed in a quiet room using a ZS-ZFT Video Analysis system before and after model establishment (ZSDC Sci-Tech Co, China). Each rat was individually placed in an opaque box (100 cm ×100 cm × 40 cm) and the bottom was divided into 25 × 25 cm^2^ equal-size squares. Rearing times and four claws climbing square numbers were considered as an index of vertical and horizontal scores, respectively. The behavior of each rat was video-recorded for 5 min [17].

Sucrose preference test was also carried out before and after model establishment. Firstly, rats were trained to adapt to 1% sucrose solution for 24 hr. Then, two bottles, one containing 1% sucrose solution and other containing tap water, were placed to each rat for 24 hr. After the adaptation, rats were deprived of water and food for 24 hr. The rats then were free to access to two bottles containing 1% sucrose solution and tap water. After 1hr, sucrose solution and tap water consumptions were measured, and the sucrose preference was calculated by the equation as follow: sucrose preference = sucrose consumption/ (sucrose consumption + water consumption) ×100% [33].

Before and after the model establishment, blood samples were collected from rats’ eye canthus and centrifuged to obtain plasma at 3,000 × g for 5 min. The plasma cortisol (CORT) and epinephrine (NE) levels were assayed with ELISA Kit according to the instructions.

### Determination of plasma repaglinide concentration

After depression model establishment, the repaglinide suspension was given by gavage at 6.0 mg/kg to all rats. Blood samples were collected into heparinized tubes at 0, 0.25, 0.5, 0.75, 1.0, 2.0, 3.0, 4.0, 6.0, 8.0 hr after administration, and then were centrifuged at 3,000 × g for 5 min at 4°C. The plasma samples were stored at –80°C for LC-MS/MS analysis.

The plasma concentration of repaglinide was determined by LC-MS/MS based on reference with a minor modification [34]. A volume of 100 μL nateglinide, as an internal standard, and 300 μL methanol were mixed with 100 μL plasma sample. Then mixture solution was vortex-mixed for 1min, followed by centrifugation at 15,000 rpm for 5 min. The supernatant was directly injected for LC-MS/MS analysis. The LC-MS/MS system consisted of Agilent 1260 HPLC (Agilent, USA) couple to an Agilent 6420 triple quadrupole mass spectrometer (Agilent, USA). The separation was performed on a ZORBAX EP- C_18_ column (50 mm × 2.1 mm, 1.8 μm, Agilent) at 35°C with methanol - 0.1% formic acid aqueous solution (80:20) as a mobile phase at a flow rate of 1.0 ml/min. The mass spectrometer was run in operated positive electrospray ionization (ESI) mode, with the electrospray voltage, gas pressure and temperature set to 4,000 V, 15 psi and 350°C, respectively, which was set to monitor the m/z 453.2 → m/z 230.1 for repaglinide and m/z 316.0 → m/z 168.1 for nateglinide, respectively. The HPLC system and mass spectrometer were controlled by Masshunter Workstation software (version B.06.00, Agilent, USA), and data were collected with the same software. Pharmacokinetic parameters were calculated by PKSolver 2.0, which is an add-in program for pharmacokinetic data analysis in Microsoft Excel [35].

### Gene expression profiling and bioinformatics analysis

GK rats were sacrificed after the pharmacokinetic experiment was finished. Rats’ livers were collected, washed with 0.9% NaCl solution, and stored at –80°C until use. Total RNA was extracted using TRIzol Reagent following the instructions. RIN number was used to inspect RNA integrity by an Agilent Bioanalyzer 2100 (Agilent technologies, Santa Clara, CA, US). Qualified total RNA was further purified by RNeasy micro kit and RNase-Free DNase Set. Total RNA were amplified, labelled and purified by using Gene Chip 3′IVT Express Kit followed the manufacturer's instructions to obtain biotin labelled cRNA.

Array hybridization and wash was performed using GeneChip^®^ Hybridization, Wash and Stain Kit in Hybridization Oven 645 (Affymetrix, Santa Clara, CA, USA) and Fluidics Station 450 (Affymetrix, Santa Clara, CA, USA) followed the instructions. Slides were scanned by GeneChip^®^ Scanner 3000 (Affymetrix, Santa Clara, CA, USA) and Command Console Software 3.1 (Affymetrix, Santa Clara, CA, USA) with default settings. Microarray quality control assessment and data acquisition were performed with the GeneChip^®^ Operating Software (GCOS, Affymetrix). Raw data which could be available from the databases (GSE94988) were normalized by MAS 5.0 algorithm, and the differentially expressed genes (DEGs) were further analyzed. The intention DEGs were mapped to the KEGG pathway, and the significant pathways were calculated based on the location and expression level of the genes in the pathway. The interaction among up-regulated DEGs was explored using GeneSpring Software 11.0 (Agilent technologies, Santa Clara, CA, USA).

### Confirmation of mRNA expression by qRT-PCR

Total RNA was extracted using TaKaRa MiniBEST Universal RNA Extraction Kit according to the instructions. The RNA concentration was determined using NanoDrop Spectrophotometer (Thermo Scientific, USA) at an absorbance of 260 nm. For quantitative RT-PCR analysis, total RNA was transcribed to cDNA using PrimeScript RT reagent Kit with gDNA Eraser. Real-time PCR was performed with ABI7500 Real-time PCR system using SYBR green quantitative PCR master mix. To normalize the mRNA expression, the housekeeping gene glyceraldehyde-3-phosphate dehydrogenase (GAPDH) was used as an external standard. The primer sequences were shown in Table 4. The relative amount of each mRNA was calculated using the 2^-ΔΔCt^ formula [36].

**Table 4:** Primer sequence for quantitative real-time polymerase chain reaction

Gene symbol	GeneBank accession No.	Primer sequence (5′→3′)	Amplicon size (bp)
*Cyp17a1*	NM_012753.2	Forward: TCTGTGCTATCTGCTTCAACATCTC	88
		Reverse: GCATCCACGATACCCTCAGTAAA	
*Cyp3a18*	NM_145782	Forward: ACAATCCTGTCTCCAACCTTCAC	115
		Reverse: GCTCCCCTTTTGCTTCTTCTC	
*Cyp2b1/2*	NM_001134844	Forward: GGGAAAGAGGAGTGTGGAAGAA	132
		Reverse: GAGCAGATGATGTTGGCTGTG	
*Ugt1a1*	NM_001039549	Forward: TTGGTGGGATAAACTGCCTTCA	165
		Reverse: 5′-GAATTCTGCCCAAAGCCTCA-3′	
*Ugt2b1*	NM_173295.1	Forward: GCTTCTGCTCTTGCCCAAATTC	176
		Reverse: GCCTCATAGATGCCATTTGTTCC	
*Gsta5*	NM_001010921.1	Forward: CATCCATGGCTGGCTTTC	165
		Reverse: CAGCCACGGATGTGCTCAA	
*Nr1i3*	NM_001270838.1	Forward: CCTACATGTTCAAGGGCGTCATC	119
		Reverse: TGTCGAACATCGTGTTGAACCTC	
*Nr1i2*	NM_052980.2	Forward: CCTACATGTTCAAGGGCGTCATC	138
		Reverse: TGTCGAACATCGTGTTGAACCTC	
*GAPDH*	NM_017008.4	Forward: TCCTGCACCACCAACTGCTTAGC	125
		Reverse: GAGGGGCCATCCACAGTCTTCTG	

### Cell tests

Rat liver cell line of BRL 3A was purchased from the cell bank, Chinese Academy of Science. The cells were cultured in DMEM supplemented with 10% FBS in a humidified incubator with 5% CO_2_ and 95% air at 37°C. After subconfluence, the cells were seeded into either 96-well or 6-well plates at the indicated density. The culture media were replaced every 2 days.

The viability of the BRL 3A cells was quantified by using Cell Counting Kit (CCK) assay. Cells were seeded into 96-well plates and dose-response experiments started 24 hr later. BRL 3A cells were treated either with DEX, or the adrenergic receptor agonists of DEXT, PE, ISO alone, at different doses ranging from 0.625–10 μmol/L and DEX in combination with the glucocorticoid receptors antagonist of the Mifepristone (RU486) or 8-Br-cAMP (cAMP analogue) for 48 hr. Then 10 uL CCK was added to each well and incubated at 37°C for 2 hr. At last, optical densities (ODs) were measured by the spectrometric absorbance at 450nm on a microplate reader (Bio-Rad Laboratories, CA, USA). Results were plotted as percent of survival and concentration-response curves were fitted in order to determine no inhibition of cell growth value. Nontoxic drug concentration for each drug was determined. Cells were seeded into 6-wells plates and treated with DEX or the adrenergic receptor agonists alone, or DEX co-treated with 8-Br-cAMP, RU-486 for 48hr. Cells were collected for mRNA and proteins expression measurement as the liver tissue.

### Western blot assay

Protein was extracted using Beyotime Biotech Reagents (Shanghai, China). The concentrations were determined with a BCA protein assay kit. The protein sample (40 μg) was loaded on 10% Bis-Tris Gel which was then transferred to NC membranes for 60 min at 2.5 mA/cm^2^ at room temperature using a Semi-Dry blotting system (Millipore Co., Chicago, IL). Membranes were blocked with 5% none fat milk in TBS/0.05% tween (TBST) for 2 hr at room temperature, washed 3 times for 10 min in TBST, and then incubated with the primary antibodies against CAR(1:500), PXR(1:1000), Cyp3a1(1:1000), Cyp2b1/2(1:1000), Ugt1a1(1:1000), Ugt2b(1:1000), GAPDH and β-actin (1:1000) diluted in TBST respectively over night at 4°C. Anti-rabbit (diluted 1:5000 in TBST) horseradish-peroxidase-conjugated second antibodies were applied after washing the blots 3 times in TBST for 10 minutes. Chemiluminescence signal was developed using an ECL kit according to the instructions, and the band signal was detected by the FluorChem FC3 System. Band densitometry was quantified using Quantity One Analysis software (version 4.5.2, Bio-Rad Laboratories, CA, USA) and signaling was expressed as relative values as normalized by GAPDH or β-actin bands.

### Statistical analysis

For microarray data analysis, ANOVA and filtering lists by *p*-value and fold-change were performed with the GeneSpring Software. Heatmap and gene clustering analysis of the gene expression data were carried out with R and BioConductor packages. A statistical comparison of the CUMS-induced effects was made by hierarchical clustering. Statistical analysis of other data was performed with GraphPad Prism 5 (GraphPad Software, Inc., La Jolla, CA, USA). Student *t*-test was used to assess statistical significance between groups. Data were expressed as mean ± standard deviation (SD). *p < 0.05, or **p < 0.01 were considered statistically significant.
